# Microfluidic measurement of intracellular mRNA with a molecular beacon probe towards point-of-care radiation triage†

**DOI:** 10.1039/d4sd00079j

**Published:** 2024-07-02

**Authors:** Xin Meng, Kechun Wen, Jingyang Zhao, Yaru Han, Shanaz A. Ghandhi, Salan P. Kaur, David J. Brenner, Helen C. Turner, Sally A. Amundson, Qiao Lin

**Affiliations:** a Department of Mechanical Engineering, Columbia University New York NY 10027 USA qlin@columbia.edu; b Center for Radiological Research, Columbia University Irving Medical Center New York New York 10032 USA saa2108@cumc.columbia.edu

## Abstract

In large-scale radiation exposure events, the ability to triage potential victims by the received radiation dosage is crucial. This can be evaluated by radiation-induced biological changes. Radiation-responsive mRNA is a class of biomarkers that has been explored for dose-dependency with methods such as RT-qPCR. However, these methods are challenging to implement for point-of-care devices. We have designed and used molecular beacons as probes for the measurement of radiation-induced changes of intracellular mRNA in a microfluidic device towards determining radiation dosage. Our experiments, in which fixed TK6 cells labeled with a molecular beacon specific to *BAX* mRNA exhibited dose-dependent fluorescence in a manner consistent with RT-qPCR analysis, demonstrate that such intracellular molecular probes can potentially be used in point-of-care radiation biodosimetry. This proof of concept could readily be extended to any RNA-based test to provide direct measurements at the bedside.

## Abstract 2

We designed molecular beacons targeting BA*X* mRNA for intracellular fluorescence measurement.

## Graphical abstract

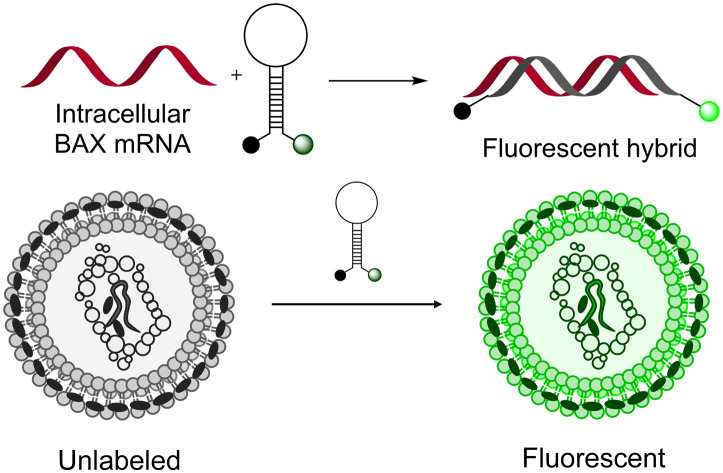

## 1. Introduction

In a large-scale radiation exposure event, hundreds of people may be exposed to varying doses of radiation. It will be crucial to identify the doses received by each individual in a timely manner to triage victims of radiation and guide medical decisions. Biodosimetry refers to the determination of radiation dosage received by an individual based on observable biological changes that occur inside the biological system. These changes are reflected in specific biomarkers, which can be used to assess the magnitude of the biological absorbed dose and inform potential consequences of the radiation exposure to the individual, thereby allowing for the administration of suitable medical therapy.^1^ To date, the dicentric chromosome assay (DCA)^2–4^ has been most used and is considered the “gold standard” for dose reconstruction accuracy. However, DCA, like all cytogenetics-based assays, is time-consuming and laborious, and does not allow timely measurement of radiation exposure in a large-scale emergency.^5^ Thus, there is a strong need for biodosimetry assays that are more rapid and of higher throughput in events of mass radiation exposure.

As an alternative method for radiation biodosimetry, additional methods such as γ-H2AX,^6,7^ micronuclei,^5,8^ microRNA,^9,10^ lncRNA,^11,12^ and protein^13–15^ and gene expression signatures^16–18^ in peripheral blood cells are being developed for this purpose.^19^ Gene expression assays have been particularly promising, and typically measure the mRNA content in a certain simulated scenario with reference to normal expression levels. Unlike DCA, gene expression can be easily assessed with advanced molecular assays and does not require cell division. Various techniques, such as real-time polymerase chain reaction (PCR)^1^ and sequencing,^20^ have been proposed for gene expression analysis. However, these methods, which require RNA to be purified from the cells for both real-time PCR and sequencing, are not suitable for translation to a rapid point-of-care (POC) assay.^21^ In comparison, analysis of gene expression at the mRNA level in whole blood cells can be highly promising for biodosimetry measurements.^22,23^

When implemented in field triage or POC scenarios, biodosimetry has the potential to guide medical decisions. POC devices have been widely reported for applications in disease diagnosis and monitoring^24–27^ based on the detection and measurement of diverse types of analytes, such as proteins,^28–30^ bacteria,^31,32^ and, in particular, nucleic acids.^33,34^ For POC analysis of nucleic acids, loop-mediated isothermal amplification (LAMP),^35^ surface acoustic wave (SAW),^36^ or CRISPR/Cas12a-based electrochemical DNA detection^37^ methods have been employed to evaluate extracellular or cell lysate samples. However, these nucleic acid analysis methods are not amenable to implementation for intracellular biodosimetry *in situ*, *i.e.*, examining the analytes at the exact location where they reside. For POC biodosimetry, Balog *et al.* reported a protein panel-based assay in non-human primate plasma samples,^38^ Brengues *et al.* reported an RNA signature using a quantitative nuclease protection assay (qNPA),^21^ and Huang *et al.* reported an approach for quantifying mRNAs using integrated CMOS detectors.^39^ Studies on POC biodosimetry methods have otherwise been scarce.^40^

### Theme

We report the design and use of molecular beacons for microfluidic *in situ* measurement of radiation-responsive intracellular mRNA with the goal of ultimately enabling POC radiation biodosimetry.

### *In situ* measurement enabled by molecular beacons (MB)

Gene expression-based methods that require isolation and purification of target sequences, while quantitatively accurate, are generally time-consuming and labor-intensive. In contrast, measurements using molecular beacons (MBs)^41^ do not require the purification of the target sequence and can be more readily performed *in situ*.^42^ MBs are hairpin-shaped oligonucleotide-based probes with a fluorescent reporter and a quencher on each end, which would be strongly fluorescent only when complementary target sequences are present.^43,44^ The fluorescence resulting from the hairpin structure possesses a low background, which is ideal for *in situ* measurements.^45^ We for the first time use MBs for *in situ* measurement of radio-induced changes of mRNA level by introducing the MB into fixed and permeabilized cells. This method would enable low-background *in situ* measurement of mRNA in TK6 cells, and for biodosimetry, can be used for *in situ* hybridization of target sequences in peripheral blood cells without isolation or purification.

### POC potential by microfluidic technology

Microfluidic technology can enable POC processing and analysis of large numbers of samples.^46^ Toward POC biodosimetry, we have designed a microfluidic device for single-cell fluorescence measurements. This device can isolate and retain fixed and labeled cells as single cells in microscale traps to quantify the intracellular fluorescence of individual cells. The single-cell device can resolve complex fluorescent signals instigated by multilayered and overlapping cells to improve the accuracy of fluorescence quantification and be used as the measurement module in POC biodosimetry.

As a proof of principle for a biodosimetry POC, we have developed MBs for a known radiation responsive biomarker BAX that will be integrated into the microfluidics system. Using the MBs, fluorescence intensities measured for TK6 cells 6 h post-exposure to radiation doses at 1 Gy and 2 Gy were respectively, 1.33-fold and 1.79-fold when compared with those of unirradiated samples. In 24 h post-exposure samples, the comparisons correspondingly became 1.45-fold for radiation at 1 Gy and 2.32-fold for radiation at 2 Gy, respectively. These changes were confirmed by experiments with NH32 p53-null control groups and found to be consistent with results from RT-qPCR quantification. Fluorescent quantification from the microfluidic device was also consistent with results obtained off-chip, suggesting the potential of the MB-based microfluidic approach for POC biodosimetry.

## 2. Results and discussion

### 2.1. Design and characterization of *BAX* MBs

#### Design of *BAX* MBs

**Fig. 1:**
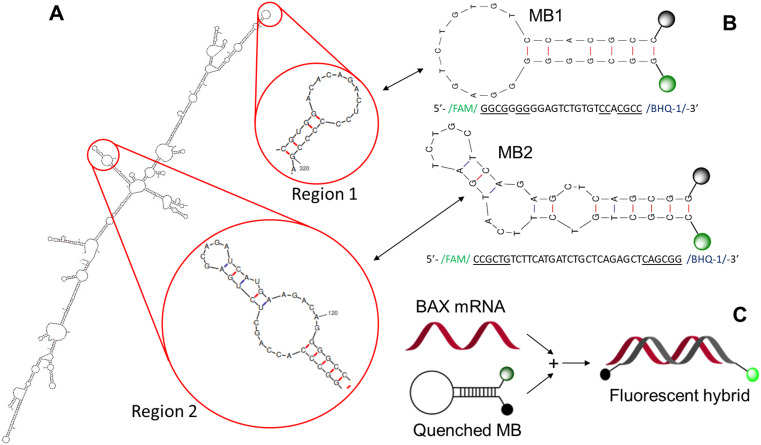
Design of *BAX* mRNA molecular beacons. (A) *BAX* mRNA secondary structure predicted by mFOLD^47^ and the two stem–loop regions selected for MB design. (B) *BAX* MB designs and sequences. (C) *BAX* MB working principle. The fluorescence of folded *BAX* MB is unquenched when it binds with the target complementary sequence.

Two potential *BAX* MBs were designed based on two regions of the predicted structure of their intended target, *BAX* mRNA. First, the secondary structure of the full-length *BAX* mRNA was predicted by mFOLD.^47^ With the predicted secondary structures, the one with the lowest Gibbs free energy was chosen and from this structure, two regions with an appropriate-sized (10–20 nts) loop sub-structures were identified and use as the initial template for MB sequence design (Fig. 1A). Using the sequences of these two regions, the reverse complements were generated as the core sequences of the *BAX* MBs. The stem sequences were also designed using the peripheral sequences of the target loop regions to increase the specificity of the MBs. The designed stem has 6 base pairs, making them fairly easy to open when targets are present, but still remain closed when target is absent. The reporter fluorophore carboxyfluorescein (FAM) was then modified to the 5′ end and its quencher Black Hole Quencher 1 (BHQ-1) to the 3′ end (Fig. 1B) to ensure the closed MB is maintained in a quenched state to minimize background signal. FAM has an emission max of 520 nm. When in proximity, the emission energy of FAM can be absorbed by BHQ-1 and emitted in the form of heat. This interaction between FAM and BHQ-1 would drastically decrease the background fluorescence of the MB when it's in the closed form. In target-free conditions, the stem–loop configuration is stable, and the quencher locates close to the reporter dye, thus quenching the reporter fluorescent signals. However, when *BAX* mRNA is present, the stem–loop configuration opens, and the MB hybridizes with the target as shown in Fig. 1C, restoring the reporter fluorescent signals. The specificity of these sequences were ensured by running through BLAST^48^ within the species genome.

#### *In vitro* characterization of *BAX* MB

**Fig. 2:**
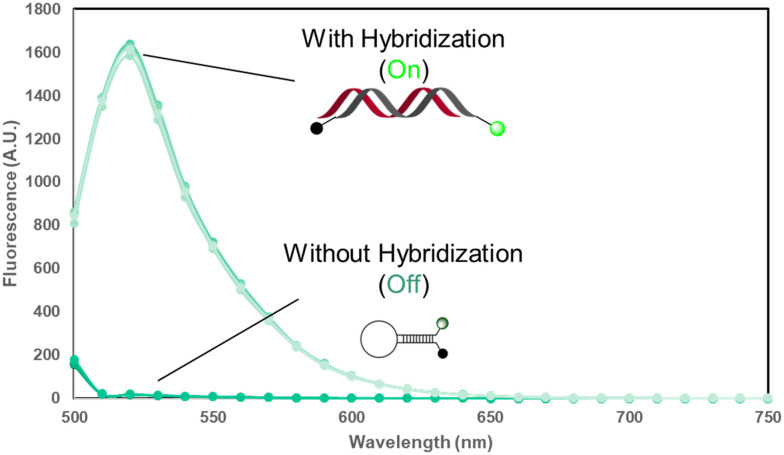
*In vitro* characterization of *BAX* MB1. Two different binding states are compared using fluorescence emission spectra. With the addition of the target sequence in DNA form, MB1 unfolds and hybridizes with the complementary sequence and a significant increase in the fluorescence spectra is present on the fluorescence spectra. The experiments were performed in triplicates for both binding states and all six fluorescence spectra were superimposed in this single figure.

To assess the ability of the MB to switch off and on, an *in vitro* hybridization experiment was designed to evaluate two of our MBs. In this experiment, the two designed MBs were incubated with their respective target sequences in the DNA form *in vitro*. In doing so, if the fluorescence is increased at FAM's intended emission spectra, it can be concluded that the self-quenched MBs are indeed switching on when encountering their target. As suggested by results in Fig. 2, MB1 shows a significant increase in fluorescence, especially at 520 nm, which is FAM's emission max. This result indicated that the design of *BAX* MB1 for its switching function is successful. The on/off fluorescence ratio for MB1 was also calculated to be ∼200 fold, indicating our MB1 design was successful.^49^ However, MB2 does not pass the *in vitro* characterization with an on/off fluorescence ratio of ∼8 fold. Because the difference in the MB behaviors of MB1 and MB2, it is suggested that MB1 would have a better performance intracellularly. We then exclusively used MB1 as the probe for the following studies.

### 2.2. Intracellular studies of *BAX* MB

#### Post-irradiation time-lapse study of MB fluorescence

**Fig. 3:**
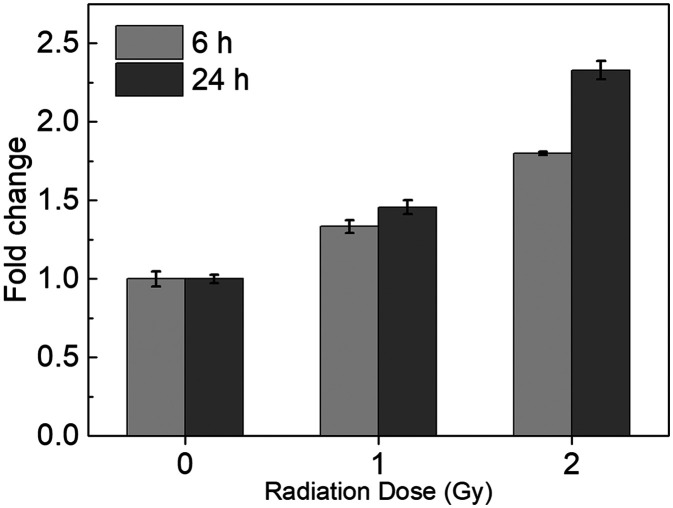
Dose-dependence of MB1 labeling with 6 h or 24 h post-exposure incubation time. All measurements were performed in triplicate and the data were shown as the mean ± SD.

The potential utility of the MBs in biodosimetry *via* measurement of radio-induced change in the abundance of intracellular mRNA was then demonstrated. To this end, dose-dependence of MB fluorescence in TK6 cells was examined. TK6 cells were first cultured to log phase and then subjected to X-ray irradiation. Samples were acquired by imaging flow cytometry and performed in triplicate on cells at 6 h after exposure to X-ray irradiation at 0, 1 and 2 Gy (Fig. 3A) to access the fluorescence intensities that reflect the amount of *BAX* mRNA within the cells. It was observed that compared to the unirradiated group, the average fluorescent intensity per cell increased almost linearly to 1.33-fold in the 1 Gy group, and 1.79-fold in the 2 Gy group, suggesting that the amount of *BAX* mRNA increases linearly with radiation dosage. Our findings of *BAX* mRNA abundance increase are consistent with previous work.^1^

Similarly, flow cytometry analysis of *BAX* mRNA levels in TK6 cells was then performed in triplicate at 24 h post-exposure to X-ray, and the same three irradiation doses (0, 1, and 2 Gy) were used (Fig. 3). Compared to unirradiated samples, the 1 Gy group showed increased fluorescence of 1.45-fold, while the 2 Gy group had increased fluorescence of 2.32-fold. The 24 h post exposure fluorescence changes were consistent with the 6 h post exposure samples at 1 Gy, and slightly higher at 2 Gy. Irradiation at 0.5 Gy was tested in a separate batch of samples (Fig. S1†). Significant changes in fluorescence were detected (1.23-fold compared to that for unirradiated cells) in TK6 cells 24 h post-exposure, suggesting that this method can detect doses as low as 0.5 Gy.

The observed upregulated BAX gene response may be mainly due to activation of the p53 signal pathway, as this is one of the main responses to radiation, and *BAX* is a known p53-regulated gene.^50^ BAX is considered an apoptosis regulator, and the upregulation of *BAX* might indicate possible apoptosis response to radiation. To further investigate the source of the increase, MB1 labeling in NH32 cells was performed. NH32 is a TK6-derived, p53 double-knockout cell line.^51^ If the radiation-induced intracellular p53 increase is the main contributor for the increased levels of *BAX*, then the irradiated group of NH32 cells would not have such a significant increase in the fluorescence. To this end, NH32 cells were cultured and irradiated at 0.5 and 2 Gy, and cultured for 24 h before cell fixation.^52^ After permeabilization, the cells were stained with MB1 and imaging flow cytometry was performed on the stained cells. The results with NH32 show that compared with the control group, the 0.5 Gy-irradiated cells have a signal of 1.01-fold, while the 2 Gy group has a signal of 1.25-fold indicating that our hypothesis regarding p53 is valid. The signal in 0.5 Gy group is essentially unchanged, suggesting that the changes in TK6 cells at 0.5 Gy are coming from the p53 pathway. The 2 Gy has a slight increase in the signal. However, comparing to the 1.73-fold change in TK6 cells, it can still be concluded that the majority of the increase in TK6 cells results from the p53 pathway.

#### Radiation dose–response comparison with RT-qPCR

**Fig. 4:**
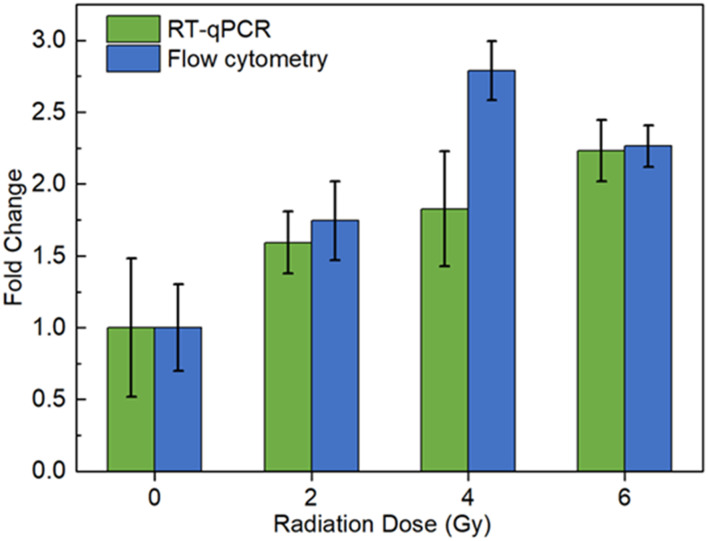
Comparison between MB labeling (imaging flow cytometry) with RT-qPCR for intracellular *BAX* mRNA biodosimetry. All measurements were performed in duplicate and the data are shown as the mean ± SD of three independent experiments.

Next, a comparison experiment was performed to assess the validity of the MB labeling signal and its correlation with actual mRNA concentration. Reverse transcription-quantitative polymerase chain reaction (RT-qPCR) is the gold standard for mRNA measurement,^53^ and was used here to compare the relative expression levels of the RNA of interest. Compared with *in situ* fluorescence measurement, RT-qPCR requires the purification of total RNA in cell lysate. The total RNA is then converted to cDNA *via* reverse transcription, before quantitative PCR is performed using specific primers for *BAX* and a fluorescent DNA dye. Compared with RT-qPCR results, the flow cytometry analysis showed similar fold changes at 2 Gy and 6 Gy (Fig. 4). The difference at 4 Gy can be explained by the variance of signals between two methods, but the results reported here are still within the previously reported range of radiation response variations.^54^ The comparison suggested that the analysis of MB fluorescent labeling by flow cytometry is valid for *BAX* mRNA detection and can be used in future experiments.

### 2.3. Microfluidic measurement of intracellular *BAX* mRNA radiation response

#### Microfluidic device design and characterization

**Fig. 5:**
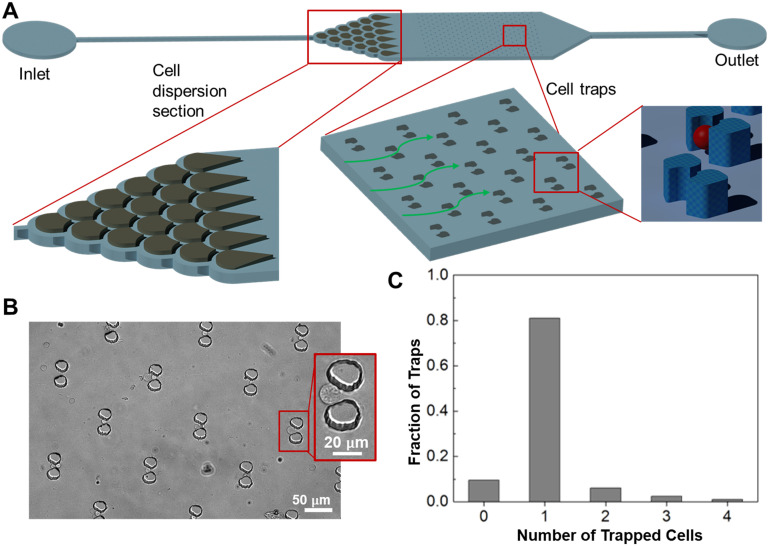
Microfluidic cell detection chamber design and cell trapping. (A) The design of cell detection chamber with blowups of the dispersion section and trapping section. (B) Image of cell-trapping units with a blowup of a trapped cell. (C) Number of cells trapped in each unit.

To further develop the utility of the MB labeling in a POC setting, the irradiated and labeled cell samples were then analyzed using a microfluidic detection device. The microfluidic device, fabricated from polydimethylsiloxane *via* standard microfabrication techniques, consists of a cell-dispersion section and a cell-trapping section located inside a microchamber (Fig. 5A). Cell samples are flowed through the microchamber, first becoming dispersed in the dispersion section and then trapped as single cells in the trapping section. The dispersion unit consists of a symmetric microchannel network about the streamwise vertical center plane of the microchamber. Cells introduced through the inlet channel are guided through the microchannel network with minimal velocity losses, and emerge from the exit of the dispersion section as uniformly distributed single cells. These cells then enter the trapping section, which consists of an array of pairs of microposts. Each micropost pair forms a microstructure having the shape of a cup with a slit at the downstream side. Single cells are directed, by the design of the cup placement, evenly into the individual cups. As the media flows through the slit, the cells are trapped in the cups.

The ability of the microfluidic device to trap individual cells was then tested. A suspension of live cells from the CCRF-CEM leukemia cell line at a concentration of 1 × 10^6^ cells per mL was flowed through the device at 5 μL min^−1^ for 5 min. The device was then washed with PBS buffer at 10 μL min^−1^ for 2 min. Images of the cell-trapping unit were finally taken to examine the trapped cells (Fig. 5B) and used to determine the fraction of cups trapping *n* cells, with *n* = 1 corresponding to trapped single cells (Fig. 5C). It was observed that the cups each trapped *n* = 0–4 cells with 80% of the cups each trapping a single cell (*n* = 1). Meanwhile, we observed that multiplying trapped cells allowed reliable quantification of fluorescence per cell (below), making our measurement method less dependent on single-cell trapping.

#### Microfluidic measurement of intracellular *BAX* mRNA dose response in TK6 cells

**Fig. 6:**
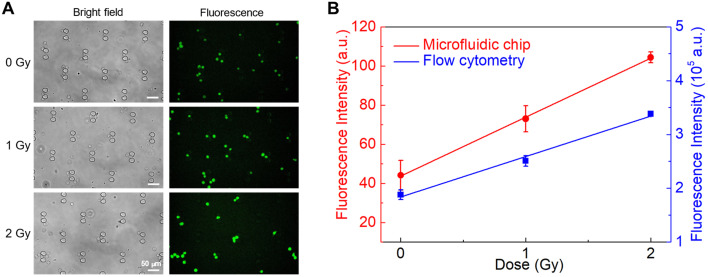
Microfluidic detection of *in situ* intracellular *BAX* mRNA dose response. (A) Bright field and fluorescence images of irradiated cell samples trapped in the microfluidic detection chamber. (B) Dose-dependence comparison of flow cytometry and microfluidic detection chamber analysis. All measurements were performed in triplicate and the data were shown as the mean ± SD.

We then tested our MB probes for measurement of intracellular mRNA in the microfluidic device. Following the same cell preparation procedure described above, TK6 cells were first exposed to X-ray radiation at 0, 1, and 2 Gy, respectively. After fixation and permeabilization at 6 h post-exposure, these cells were incubated with the *BAX* mRNA-targeting probe MB1. The cells were introduced into the microfluidic device and the trapped cells were imaged to obtain the average fluorescence intensity (Fig. 6A). The flow cytometry measurement results from Fig. 6A, while not to be quantitatively compared to the microfluidic imaging data, are included in Fig. 6B for qualitative comparison. The fluorescence intensity determined from the microfluidic device increased linearly with the irradiation dose, exhibiting the same trend as that observed from flow cytometry above and reflecting radiation-induced upregulation of intracellular *BAX*. Thus, we have successfully demonstrated that our microfluidic approach is a potentially viable method for intracellular biomarker measurements in POC radiation biodosimetry.

## 3. Experimental

TK6 and NH32 cells are gifts from Dr. Sally Amundson. CCRF-CEM cells are purchased from ATCC. Nucleic acids are purchased from Integrated DNA Technologies. All the other reagents unless otherwise specified are purchased from BD Biosciences.

### *In vitro* folding assay

The assay was performed in 96-well plates and detected with Molecular Devices SpectraMax M5 plate reader. In 250 μL microtubes, the molecular beacons (2 μM in PBSM, 50 μL) were mixed with their intended target sequence in DNA form (2 μM in PBSM, 50 μL) to reach a final concentration of 1 μM of each species. The mixture was then conditioned (10 min at 95 °C, 10 min at 0 °C and 10 min at RT) before being transferred into a 96-well plate and analyzed in a plate reader.

### Cell culture

TK6, NH32 and CCRF-CEM cell lines were maintained using culture media consisting of RPMI 1640 1× medium, 10% fetal bovine serum, 1% MEM non-essential amino acids, 1 mM sodium pyruvate and 1% penicillin–streptomycin–glutamine in a humidified 37 °C incubator with 5% CO_2_. Cell density was kept below 1 × 10^6^ cells per mL at all times. Once the cells approached 1 × 10^6^ cells per mL, a 1 : 50 dilution was performed. All cell-related experiments were performed using cells within 25 passages.

### Irradiation

Mock and the irradiation of the cell line samples was performed on an X-RAD 320 Biological Irradiator (Precision X-Ray Inc.) at 320 mV, 12.5 mA, with a custom filter^13^ and a 40 cm sample distance to achieve 1 Gy min^−1^ of irradiation rate. All samples were irradiated under the same conditions with various irradiation times.

### Cell fixation

Cell fixation was performed according to our previously published method.^15^ TK6 cell samples (<15 mL each) were centrifuged at 200 × *g* for 10 min and the supernatants were discarded. The pellets were resuspended in 250 μL of ice-cold Fixation/Permeabilization Solution (Cytofix/Cytoperm™ Fixation/Permeabilization Solution kit, BD Biosciences; #554714) and incubated at 4 °C for 20 min to allow cell fixation. After the addition of 700 μL of 1 × Perm/Wash buffer (BD Biosciences; #554723), the suspensions were centrifuged at 300 × *g* for 4 min and the supernatant removed. The pellet was then washed twice with 950 μL of 1 × Perm/Wash buffer before being resuspended with 1 mL of 1% (m/v) bovine serum albumin solution in 1 × DPBS and preserved at 4 °C.

### Cell labeling

Cell labeling with MB1 was adapted from our earlier work.^15,52^ Briefly, fixed TK6 cells preserved in 1% BSA buffer were first centrifuged at 300 × *g* for 4 min and the supernatant was discarded. The pellet was washed with 950 μL of 1 × Perm/Wash buffer The MBs were diluted in 1 × Perm/Wash buffer and conditioned in the following order: 10 min at 95 °C; 10 min on ice; 10 min at RT. The pellet is then resuspended in 50 μL of the conditioned aptamer solution and incubated for 1 h at RT away from light. After incubation, 900 μL of 1 × Perm/Wash buffer was added to the suspension, and the sample was then centrifuged at 300 × *g* for 4 min. After discarding the supernatant, the pellet was washed once again with 950 μL of 1 × Perm/Wash buffer and twice with 950 μL of 1 × PBS. If not directly used in the following fluorescence analysis, the pellet was then resuspended in 1 mL of 1 × PBS and stored at 4 °C.

### Imaging flow cytometry

Samples were concentrated to 50 μL and acquired on ImageStreamX MkII at Columbia University Center for Radiological Research with 40× objective and 488 nm excitation laser set to 200 mW. Similar to previously described,^52^ a uniform analysis template on Image Data Exploration and Analysis Software (IDEAS®, Luminex ver. 6.2) was used to measure Mean Fluorescence Intensity detected on the 480–560 nm channel in focused, single, healthy cells.

### RNA isolation and real-time PCR

At 24 hours after irradiation, whole-cell RNA was isolated from aliquots of at least 1 × 10^6^ exponentially growing cells per point using the Qiagen RNeasy Mini kit (cat#74106), and quantified using a Nanodrop One spectrophotometer (Thermofisher). The *A*_260/280_ ratios for all samples ranged from 1.97–2.03. For each sample, cDNA was prepared from 1 mg of total mRNA using the High-Capacity® cDNA kit (Life Technologies, Foster City, CA). Quantitative real-time RT-PCR (qRT-PCR) was then performed using 15 ng of cDNA per reaction and Taqman® assays (Life Technologies) for BAX (Hs00180269_m1) and the housekeeping gene ACTB (Hs99999903_m1). All reactions were performed in duplicate on a QuantStudio™ 7 Flex System, using standard PCR conditions. Expression Suite software ver. 1.3 (Thermofisher) was used to calculate relative fold-induction with the 2^−ΔΔCT^ method.

### Microfluidic device fabrication and packaging

The PDMS mold was fabricated with various SU-8 photoresists. 33 mL of PDMS (10 : 1 base : curing agent by weight) was poured onto the flow layer wafer and placed in a vacuum desiccator for 30 minutes to remove bubbles. Following bubble removal, the flow layer wafer was placed on a hotplate set at 72 °C for 30 minutes. The PDMS was then peeled off, cut into separate microfluidic chambers and had inlets and outlets punched with an autopsy punch. The PDMS microfluidic chambers and glass slides were then placed in an oxygen plasma etcher (Diener Plasma Etch) and exposed to oxygen plasma for 45 seconds at 100% power. Immediately after exposure, the PDMS devices were bonded with the glass slide on the reactive interface and gently pressed to put their surfaces in contact.

## 4. Conclusion

In summary, we described the development of a DNA molecular beacon that can bind specifically with intracellular human *BAX* mRNA towards POC radiation triage application. The design is based on a step-loop region of the predicted secondary structure of human *BAX* mRNA, and the sequence specificity is verified through BLAST. We demonstrated the unfolding of the MB probe in the presence of the target sequence *in vitro*. The MB fluorescence signal shows dose-dependence at both 6 h and 24 h post-exposure time, and up to 6 Gy of irradiation in fixed TK6 cells. The MB labeling is comparable with RT-qPCR quantification in terms of fold change. A microfluidic intracellular biodosimetry device was designed and fabricated to trap single fixed cells and we compared the on-chip quantification with flow cytometry analysis and the results are comparable. It can be concluded that the MB probe for *BAX* mRNA is suitable for microfluidic intracellular biodosimetry.

We envision the prototype device developed in this manuscript as a pivotal component of a prospective point-of-care device for integrated intracellular biodosimetry. The integration of the MB probe into a point-of-care device would enable real-time, on-site assessment of radiation exposure, thereby making biodosimetric assessments more accessible and timelier. This could be especially invaluable in emergency scenarios where rapid evaluation and immediate medical intervention are critical for optimizing patient outcomes.

## Data availability

Data for the article cannot be made publicly available due to legal requirements.

## Conflicts of interest

There are no conflicts to declare.

## Supplementary Material

SD-003-D4SD00079J-s001
