# Supplementary material for: Microfluidic measurement of intracellular mRNA with a molecular beacon probe towards point-of-care radiation triage
Source: Sens Diagn. 2024 Jul 2;3(8):1344–52. doi: 10.1039/d4sd00079j (PMC11308381; doi:10.1039/d4sd00079j)
Supplement: SD-003-D4SD00079J-s001 [file SD-003-D4SD00079J-s001.pdf]

Electronic Supplementary Material (ESI) for Sensors & Diagnostics.
This journal is © The Royal Society of Chemistry 2024

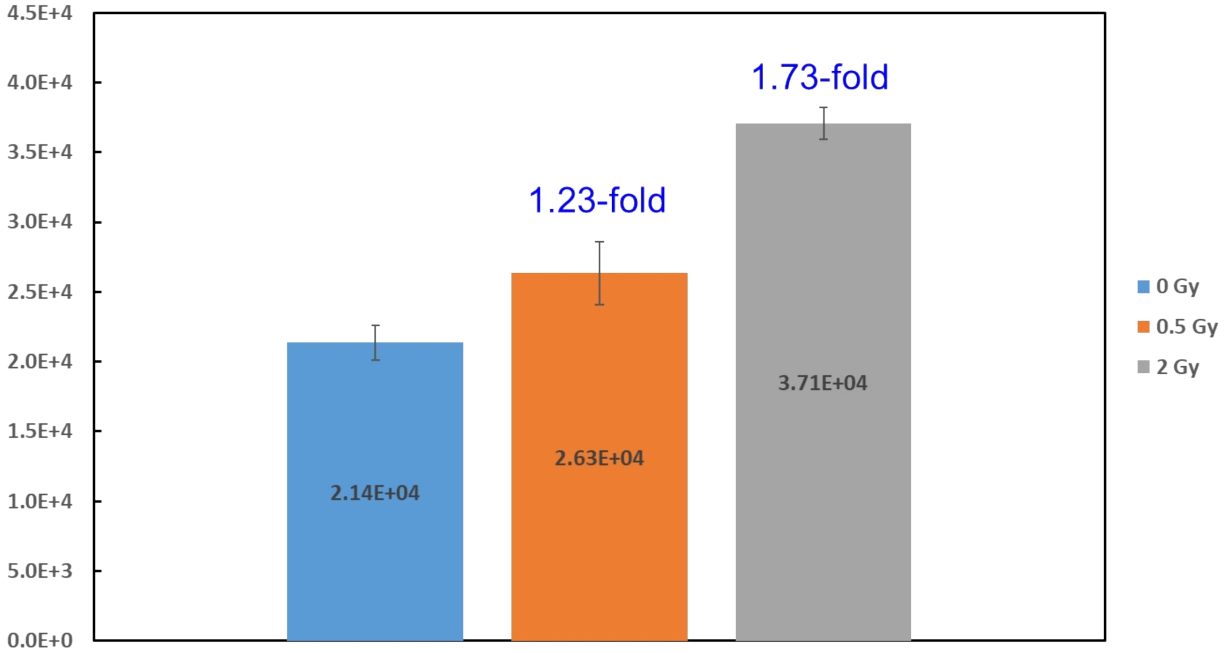

Figure S1. Dose-dependence Response of MB1 labeling with 24-h post-exposure incubation time in TK6 cells with a low dosage range.

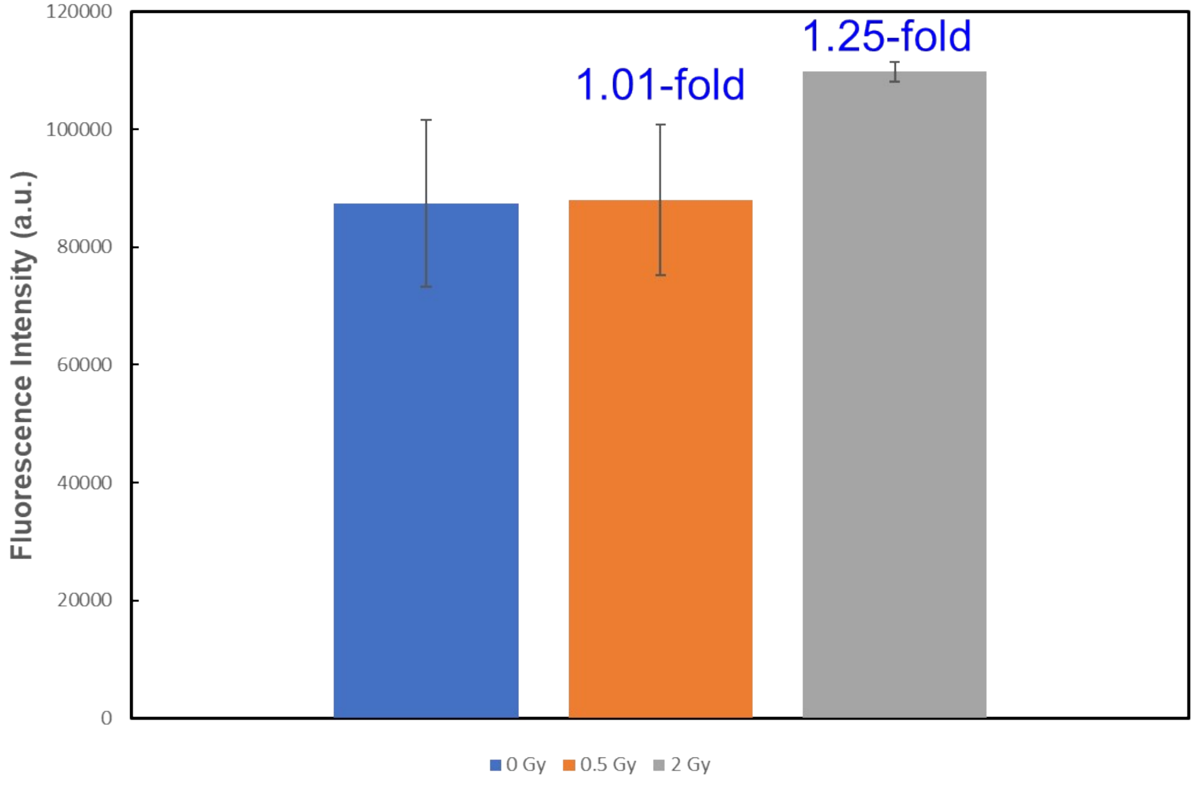

Figure S2. Dose-dependent Response of MB1 labeling with 24-h post-exposure incubation time in NH32 cells with a low dosage range.

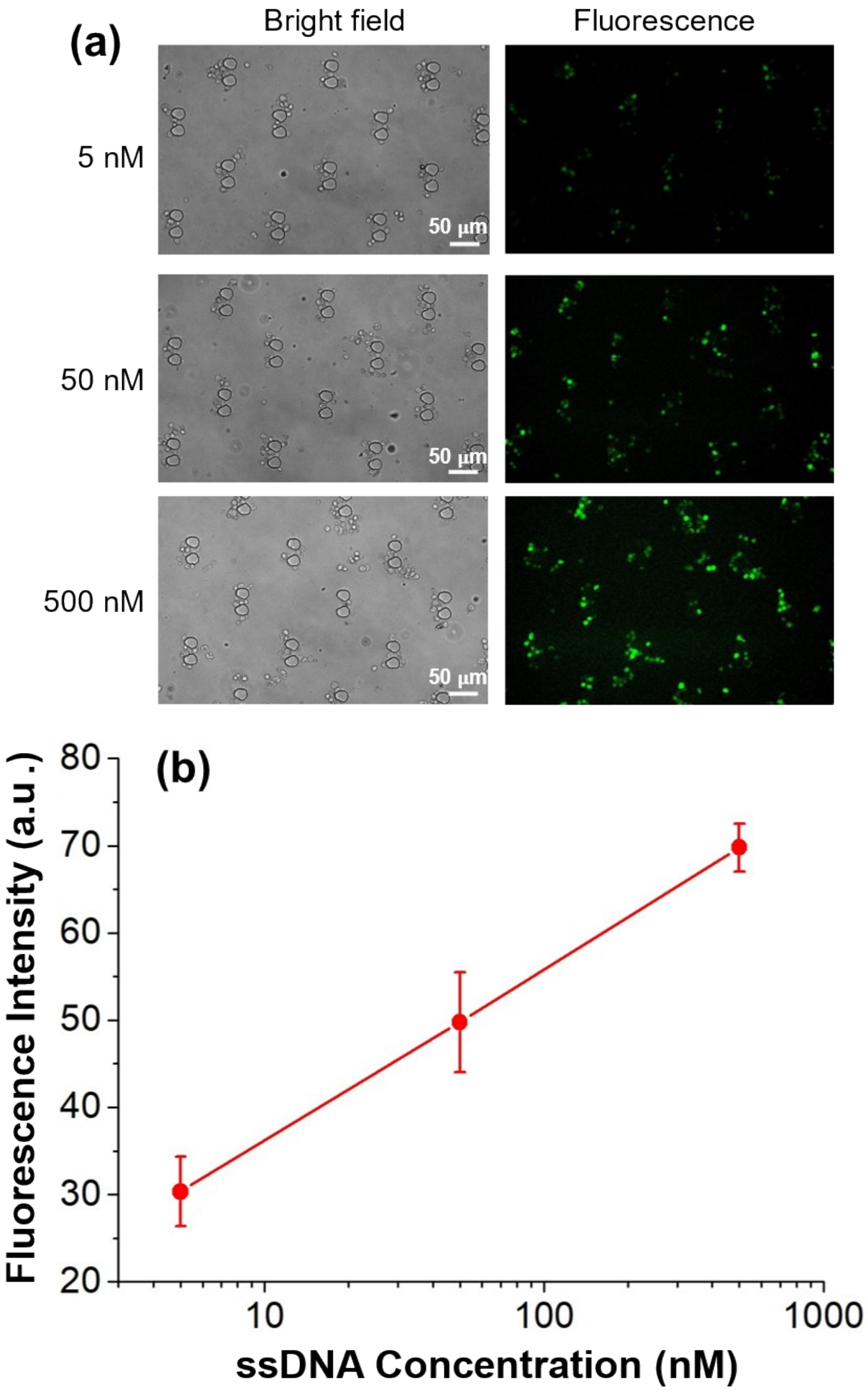

Figure S3. On-Chip Fluorescence-Concentration Correlation with Small Fluorescence Tag. (A) Fluorescent microscopy images. (B) Quantification of fluorescence.
